# Transcriptome Reveals the Regulation of Exogenous Auxin Inducing Rooting of Non-Rooting Callus of Tea Cuttings

**DOI:** 10.3390/ijms25158080

**Published:** 2024-07-24

**Authors:** Shuting Wang, Huanran Wu, Yazhao Zhang, Guodong Sun, Wenjun Qian, Fengfeng Qu, Xinfu Zhang, Jianhui Hu

**Affiliations:** College of Horticulture, Qingdao Agricultural University, Qingdao 266109, China; wangst_ang@163.com (S.W.); gentlewhr@163.com (H.W.); zhangyztea@163.com (Y.Z.); 15589206001@163.com (G.S.); qau-wenjunqian@qau.edu.cn (W.Q.); 201901153@qau.edu.cn (F.Q.); zxftea@163.com (X.Z.)

**Keywords:** *Camellia sinensis*, softwood cuttings, transcriptome, gene expression, adventitious root

## Abstract

Cuttage is the main propagation method of tea plant cultivars in China. However, some tea softwood cuttings just form an expanded and loose callus at the base, without adventitious root (AR) formation during the propagation period. Meanwhile, exogenous auxin could promote the AR formation of tea plant cuttings, but the regulation mechanism has not yet explained clearly. We conducted this study to elucidate the regulatory mechanism of exogenous auxin-induced adventitious root (AR) formation of such cuttings. The transcriptional expression profile of non-rooting tea calluses in response to exogenous IBA and NAA was analyzed using ONT RNA Seq technology. In total, 56,178 differentially expressed genes (DEGs) were detected, and most of genes were significantly differentially expressed after 12 h of exogenous auxin treatment. Among these DEGs, we further identified 80 DEGs involved in the auxin induction pathway and AR formation. Specifically, 14 auxin respective genes (ARFs, GH3s, and AUX/IAAs), 3 auxin transporters (AUX22), 19 auxin synthesis- and homeostasis-related genes (cytochrome P450 (CYP450) and calmodulin-like protein (CML) genes), and 44 transcription factors (LOB domain-containing protein (LBDs), SCARECROW-LIKE (SCL), zinc finger protein, WRKY, MYB, and NAC) were identified from these DEGs. Moreover, we found most of these DEGs were highly up-regulated at some stage before AR formation, suggesting that they may play a potential role in the AR formation of tea plant cuttings. In summary, this study will provide a theoretical foundation to deepen our understanding of the molecular mechanism of AR formation in tea cuttings induced by auxin during propagation time.

## 1. Introduction

The tea plant (*Camellia sinensis* (L.) O. Kuntze.) has been cultivated for thousands of years in China. Currently, the tea plant is one of the main economical crops in many areas of China. As the largest tea cultivation area and highest tea-producing country, it is essential to improve the propagate efficiency and then cultivate more excellent tea plant cultivars to match and meet the different needs of domestic and foreign markets, which is deeply valued by the government, tea companies, and tea researchers. In recent years, many researchers have focused on exploring how to improve the propagate efficiency of tea plants [1,2,3,4,5]. Currently, it is agreed that the vegetative propagation method is the most important path for tea plant propagation, as the offspring will maintain the same characteristics of the parents for a long time. Vegetative propagation includes cuttings, grafting, striping, and tissue culture, in which cuttage has a higher reproduction coefficient than engraft and layering, etc., and also has a relative high survival rate and a relative low cost. At present, cuttage is widely used in tea industry.

It is well known that the growth and development of adventitious roots (ARs) are the key step for successful cutting propagation. AR formation is a complex developmental process, which is faced with many stress conditions and regulated by many factors [6,7]. Many researchers have reported that exogenous auxin could affect endogenous hormone levels, as a result of the changes in plant developmental processes [8,9]. In tea plants, RNA-Seq analysis of IBA-treated or non-IBA-treated cuttings revealed that auxin homeostasis-related genes were clearly affected [10]. However, IBA pre-treatment slightly alters the expression of a gene encoding an auxin efflux carrier (*MiPIN1*) but significantly induces the expressions of two genes encoding auxin influx carriers (*MiAUX3* and *MiAUX4*) in mango cotyledon fragments [11]. In addition, many studies have found that auxin response factor (ARF)-relative TF genes regulate the expression of auxin-responsive genes, e.g., *GH3*, at the transcriptional level [12,13]. *GH3* is involved in the binding of free auxin to amino acids, and overexpression of *GH3* results in auxin deficiency in plants [14]. Plant-specific lateral organ boundary domain (LBD) TFs act downstream of the ARF and mainly modulate AR formation by regulating cell division and cell wall modification during AR generation [15]. In Arabidopsis, SCARECROW (SCR) interacts with SHORTROOT (SHR) to activate downstream target genes, thereby regulating root apical meristem (RAM) [16]. During AR initiation, auxin induced WUSCHEL-related homeobox 11 (*WOX11*) in AR founder cells, and then *WOX11* activated the expression of *LATERAL ORGAN BOUNDARIES DOMAIN 16* (*LBD16*) [17]. The overexpression of a WOX5 gene from Liriodendron hybrid (LhWOX5) affected the apex meristem morphology, resulting in a curled and shortened taproot in transgenic Arabidopsis [18].

Single-node cutting and softwood cutting are the two most commonly tea cutting breeding method. For single-node cutting, it is necessary to grow 20–30 cm of new shoots before they can emerge from the nursery after the root system is formed, so it takes more than one year to transplant from the nursery. Compared with single-node cutting, softwood cutting has a shorter seedling raising cycle; once the root system is formed, it reaches the nursery standard, which has been widely used and popularized in Shandong tea area [19]. However, some softwood cutting seedlings just form expanded and loose calluses at the base of softwood cuttings and not the adventitious roots during propagation period. This phenomenon greatly reduced the survival rate and quantity of tea softwood cutting seedlings in the tea industry. In 2021, the proportion of non-rooting calluses in all tea cutting callus was 40.15% for softwood cutting of *Camellia sinensis* cv. ‘ShuChaZao’ [19]. We previously found exogenous hormone treatment could promote AR formation for those tea cutting seedlings that only formed calluses without adventitious roots. However, the molecular mechanism of exogenous hormone promoting AR formation of those tea cutting seedlings has not been explored. In order to explore the induction and regulation of exogenous auxin on callus rooting, transcriptome technology was used to analyze the change in gene transcription in the basal tissue of cuttings under exogenous auxin treatment conditions. The results of this study laid a theoretical foundation for subsequent use of exogenous auxin to induce rooting in cuttings that only form rootless calluses and improve the survival rate of tea cuttings.

## 2. Results

### 2.1. Phenotypic Changes in the Roots of Cuttings Treated with Exogenous Auxin

**Figure 1:**
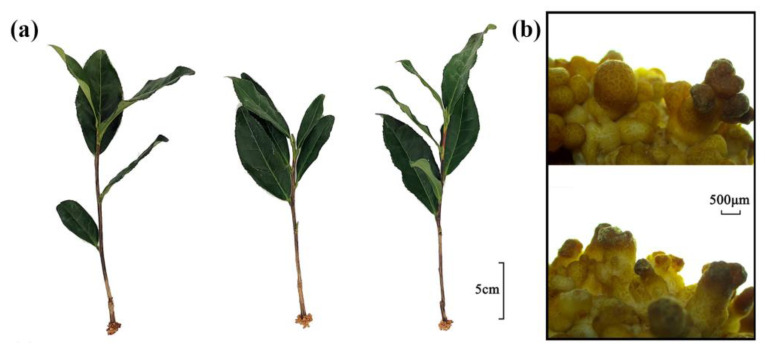
Phenotypic observation of tea plant cutting seedlings. (**a**) Phenotypic state of cuttings. (**b**) Root callus of cuttage seedlings.

The cuttings were approximately 15 cm long and a swollen callus was formed on the base incision without adventitious roots. The basal callus was light yellow in color and small irregular cracks were observed on the surface. Dense, irregular columnar protrusions were observed on the callus surface, and the top of the protrusion was brown (Figure 1a,b). The phenotype of the cuttings showed no significant change after distilled water treatment. After thirty-five days of exogenous auxin treatment, cuttings began to show adventitious roots. It indicated that exogenous auxin treatment promoted the growth of adventitious roots.

### 2.2. Full-Length RNA-seq and Functional Annotation

**Figure 2:**
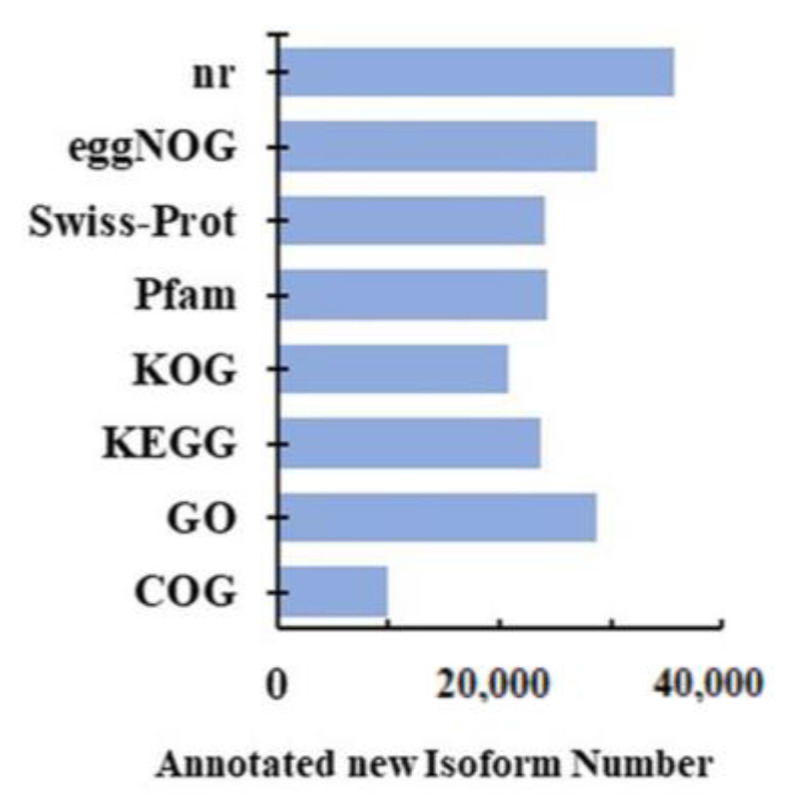
Functional annotations of new isoforms in the indicated databases. DIAMOND software was used to compare the sequences of the discovered new genes with the NR, Swiss-Prot, COG, KOG, and KEGG databases.

To clarify the molecular mechanisms of root formation of callused, rootless cuttings induced by exogenous auxin, transcripts of the swollen callus of the differentially treated cuttings were sequenced. A total of 24 cDNA libraries from eight samples, including the hormone treatment groups (HT6, HT12, HT24, and HT48 mean 6, 12, 24, and 48 h treatment time) and distilled water treatment groups (WT6, WT12, WT24, and WT48 mean 6, 12, 24, and 48 h treatment time). Three biological replicates were performed for each treatment. The average of the clean data obtained for each sample reached 450,379, and the total number of sequenced bases is already given in Table S1. From the initial 108,090,979 raw reads, 102,601,685 (94.92%) clean reads were obtained (Table S1). The cleaned sample data were aligned to the ribosomal RNA sequences in the database using the alignment tool BLAST. The ribosomal RNA was discarded after mapping to an rRNA database. After mapping to the *R. chinensis* ‘*Camellia sinensis* (Chrlev)’ genome, 56,178 genes were identified in the transcriptome. Clusters of FLNC transcripts were obtained after mapping to a reference genome with mimimap2, and consensus isoforms were obtained after polishing within each cluster by pinfish. Gffcompare (version 0.9.8, parameter-r-R) was used to compare the known annotation of the reference genome to find the original unannotated transcript regions, explore new transcripts and new genes of the species, and name the identified transcripts by ONT. Furthermore, 9935; 28,741; 23,649; 20,733; 24,222; 24,004; 28,625; and 35,667 new isoforms were annotated in the GeneOntology (GO: http://geneontology.org/ (accessed on 29 March 2023)); Kyoto Encyclopedia of Genes and Genomes (KEGG: https://www.genome.jp/kegg/ (accessed on 29 March 2023)); Clusters of Orthologous Groups of proteins (KOG: https://www.ncbi.nlm.nih.gov/COG/ (accessed on 29 March 2023); COG: https://www.ncbi.nlm.nih.gov/COG/ (accessed on 29 March 2023); eggNOG: http://eggnogdb.embl.de/ (accessed on 29 March 2023)), Protein family (Pfam: https://pfam.xfam.org/ (accessed on 29 March 2023)), a manually annotated and reviewed protein sequence database (Swiss-Prot: https://www.uniprot.org/uniprot/ (accessed on 29 March 2023)), and NCBI non-redundant protein sequence (NR: https://www.ncbi.nlm.nih.gov/ (accessed on 29 March 2023)) databases, respectively (Figure 2).

### 2.3. Comparative Analysis of DEGs

**Figure 3:**
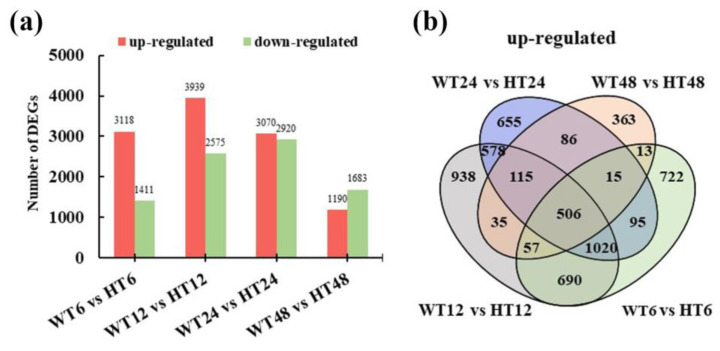
DEGs in cuttings calluses in response to auxin induction. (**a**) The number of DEGs between the hormone treatment and distilled water treatment groups in the indicated groups. (**b**) Venn diagram showing the overlapping up-regulated DEGs between WT6 vs. HT6, WT12 vs. HT12, WT24 vs. HT24, and WT48 vs. HT48.

To further elucidate the auxin-induced transcriptomic change in calluses, a total of 10,935 DEGs were detected in WT6 vs. HT6, WT12 vs. HT12, WT24 vs. HT24, and WT48 vs. HT48; differential expression analysis of two groups was performed using the DESeq2 R package (1.6.3). The resulting *p* values were adjusted using the Benjamini and Hochberg’s approach for controlling the false discovery rate. Genes with FDR < 0.01 and foldchange ≥ 2 found by DESeq2 were assigned as differentially expressed (Figure 3a). We detected 4529, 6514, 5990, and 2873 DEGs in the four pairwise comparisons, respectively. In a total, 5888 up-regulated and 5402 down-regulated DEGs were identified after hormone treatment. The expressions of large numbers of DEGs increased firstly and then decreased over time. However, the highest number of DEGs were up-regulated at the 12 h treatment time point, and the highest number of DEGs were down-regulated at the 24 h treatment time point. Furthermore, with the increase in processing time, the number of down-regulated DEGs was gradually higher than that of up-regulated DEGs. Venn diagrams were created to show the distribution of up-regulated DEGs among the four pairwise comparisons of WT6 vs. HT6, WT12 vs. HT12, WT24 vs. HT24, and WT48 vs. HT48 (Figure 3b). A total of 506 genes overlapped in all comparisons, 115 DEGs were up-regulated in WT12 vs. HT12, WT24 vs. HT24, and WT48 vs. HT48, and 1020 DEGs were up-regulated in WT6 vs. HT6, WT12 vs. HT12, and WT24 vs. HT24, in which five down-regulated DEGs in WT48 vs. HT48 were filtered. All 1636 DEGs were considered to be successfully up-regulated by exogenous auxin treatment, which might contribute to the rooting of cuttings, and they were defined as Group1 (G1) for further analysis.

**Figure 4:**
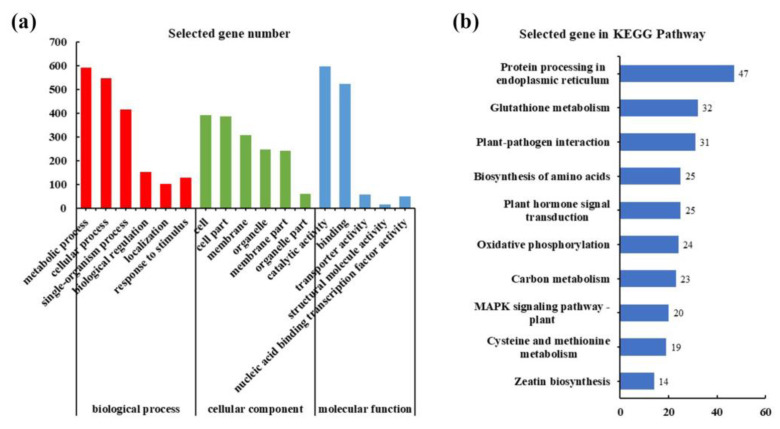
GO and KEGG annotation classification statistics plot of up-regulated DEGs. (**a**) Statistical plots of GO annotation classification of DEGs. (**b**) KEGG classification plots of DEGs, the top 10.

In order to clarify the function of the DEGs in G1, GO and KEGG enrichment analyses were performed. The results show that these DEGs annotated in the GO database were divided into several categories: biological process (the top three GO terms were the metabolic process, cellular process, and single-organism process), cellular component (cell, cell part and membrane), and molecular function (catalytic, binding, and transporter activity) (Figure 4a). Furthermore, the enrichment analysis results of the KEGG pathway showed that the DEGs were mainly enriched in the pathway of “protein processing in endoplasmic reticulum”, “glutathione metabolism”, “plant-pathogen interaction”, “biosynthesis of amino acids”, and “plant hormone signal transduction” (Figure 4b).

### 2.4. Identification of Auxin-Related DEGs

To elucidate the molecular mechanism of AR formation mediated by auxin, the DEGs with low transcriptional abundance were filtered out, and a total of 1424 DEGs were obtained from G1, which was defined as G2. Based on the gene annotation information (Table S4), we found that AR formation was mainly linked by the DEGs involved in IAA synthesis-, homeostasis-, and receptor-related processes. Then, the auxin-related DEGs were screened out from G2. As a result, fourteen auxin-induced, response-related, and transport-related protein DEGs were identified (Table 1), including nine auxin response protein genes, two auxin response factor, two auxin transport carrier genes, and one auxin-induced protein gene. Among them, the expression of *ARF5* was significantly induced after 6 h of exogenous auxin treatment, and then the expression was dramatically decreased with the treatment performed. In addition, four *GH3* family genes were differentially expressed in the four pairwise comparisons; except for a *GH3* family gene, *GH3.5* was not changed in the WT6 vs. HT6. Similarly, the expression of *IAA11* was also unchanged in WT6 vs. HT6. Meanwhile, the expressions of *ARF9* and 2 auxin transport carrier genes were up-regulated at the 6 h, 12 h, and 24 h time points, respectively.

**Figure 5:**
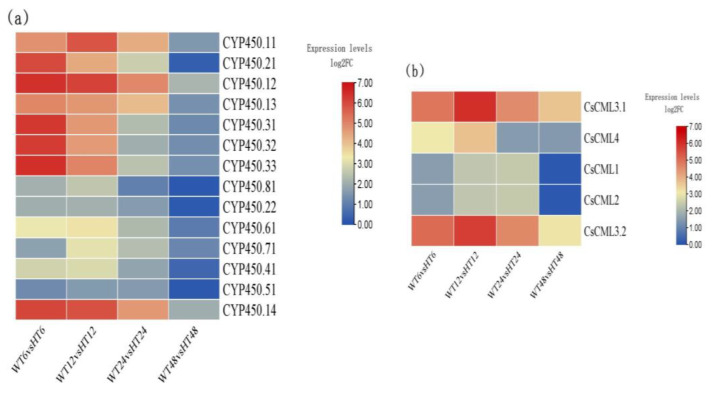
Heatmap of selected DEGs. (**a**) Heatmap of CYP450 transcription factor. (**b**) Heatmap of CML genes (HT6, HT12, HT24, and HT48 mean 6-, 12-, 24-, and 48-h hormone treatment; WT6, WT12, WT24, and WT48 mean 6-, 12-, 24-, and 48-h distilled water treatment).

During IAA synthesis, cytochrome *P450 (CYP450)* family genes and calmodulin-like protein (*CML*) genes play important roles in IAA accumulation [20]. In this study, fourteen *cytochrome P450* family genes and five *CML* genes were differentially expressed (Figure 5). Among them, some genes, such as *cytochrome P450 81E8*-*like* (*CSS0050435*, *CSS0022577*, *CSS0049182*, and *ONT*.*3537*), cytochrome *P450 94A2*-*like* (*CSS0006878*, *CSS0018287* and *CSS0029619*), and *cytochrome P450 71D10* (*CSS0013294*), were all up-regulated in WT6 vs. HT6, WT12 vs. HT12, and WT24 vs. HT24. However, after 48 h of exogenous auxin treatment, the expressions of these genes were attenuated, of which the expressions of seven *CYP450* genes and two *CML* genes were decreased to a normal level.

### 2.5. Auxin-Responsive LOB-Domain TFs and SCL Family Genes

**Figure 6:**
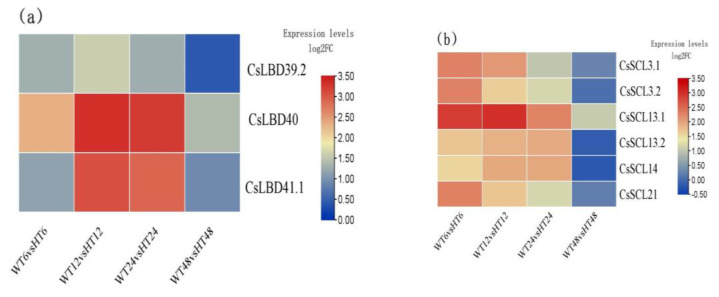
Heatmap of selected DEGs. (**a**) Heatmap of LBD transcription factor. (**b**) Heatmap of SCL genes (HT6, HT12, HT24, and HT48 mean 6-, 12-, 24-, and 48-h hormone treatment; WT6, WT12, WT24, and WT48 mean 6-, 12-, 24-, and 48-h distilled water treatment).

In this study, we found three *LATERAL ORGAN BOUNDARIES*-*DOMAIN* (*LBD*-*domain*, *LBD*) genes and six *SCARECROW*-*LIKE* (*SCL*) genes were differentially expressed in G2 (Figure 6a,b). The LBD DEGs mainly contained *CsLBD39_2* (*CSS0006814*), *CsLBD40* (*CSS0015221*), and *CsLBD41_1* (*CSS0026995*). The *SCL* DEGs mainly contained *SCL3* (*CSS0026522* and *CSS0003129*), *SCL13* (*CSS0022566* and *CSS0033997*), *SCL14* (*CSS0027812*), and *SCL21* (*CSS0020123*). Similar to the expression of the above DEGs, these nine genes were highly expressed at 6 h, 12 h, and 24 h time points, of which *CsLBD40* and *SCL21* were up-regulated in all pairwise comparisons.

### 2.6. DEGs of TF Families That Affected by Auxin Treatment

**Figure 7:**
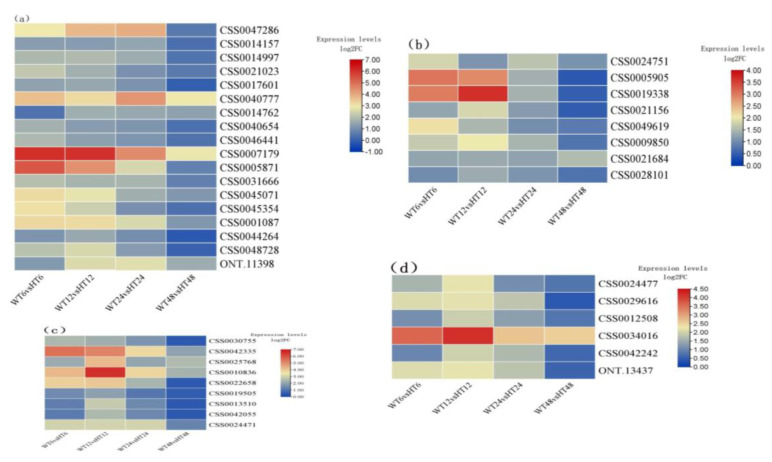
Heatmap of some TF family DEGs. (**a**) Heatmap of zinc finger TFs. (**b**) Heatmap of WRKY. (**c**) Heatmap of NAC TFs. (**d**) Heatmap of MYB TFs hormone treatment group (HT6, HT12, HT24, and HT48 mean 6-, 12-, 24-, and 48-h treatment time) and distilled water treatment group (WT6, WT12, WT24, and WT48 means 6-, 12-, 24-, and 48-h treatment time).

It is well known that many zinc finger, WRKY, MYB, and NAC TF family genes are involved in root apical meristem (RAM). Among the selected DEGs, the expressions of most of zinc finger TF genes were unchanged in WT48 vs. HT48 (Figure 7a). However, the expressions of *Zinc finger Ran*-*binding domain*-*containing protein 2-like* (*CSS0040777*), Zinc finger AN1 domain-containing stress-associated protein 12-like (*CSS00007179*), *zinc finger protein ZAT10* (*CSS0045071* and *CSS0001087*), and *FCS*-*Like Zinc finger 3*-*like* (*ONT*.*11398*) were up-regulated in the four pairwise comparisons, of which *Zinc finger AN1 domain*-*containing stress*-*associated protein 12*-*like* was significantly differentially expressed at 6 h and 12 h treatment time points. Meanwhile, the expression of *zinc finger*, *RING*/*FYVE*/*PHD*-*type* (*CSS0005871*) was also significantly differentially expressed in WT6 vs. HT6 and WT12 vs. HT12 but not changed at 48 h treatment time points.

In addition, eight WRKY, six MYB, and nine NAC TFs were differentially expressed (Figure 7b,c). Among them, WRKY 22 (CSS0024751), WRKY 23 (CSS0021684), MYB108-like (CSS0034016), NAC transcription factor (CSS0042335), NAC domain-containing protein 90-like (CSS0025768 and CSS0010836), and NAC domain-containing protein 73-like (CSS0024471) were all differentially expressed at four treatment time points, while the other differentially expressed TF genes were not changed after 48 h of treatment as compared with the control.

### 2.7. qRT-PCR Verification of Gene Expression

**Figure 8:**
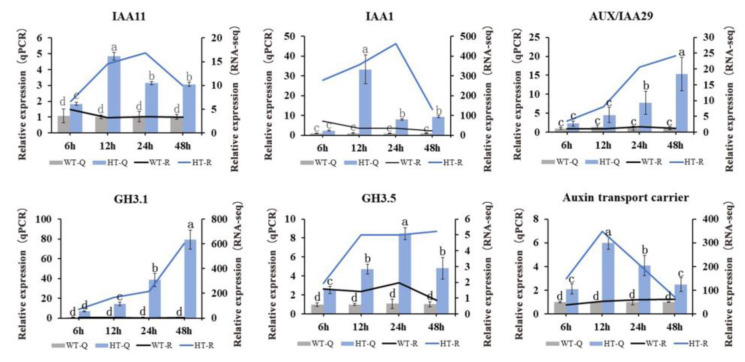
The relative expression levels of six selected DEGs were compared by RNA-seq (R) and qRT-PCR (Q). Relative expression levels obtained from qPCR and RNA-Seq (CPM) are shown as column and line respectively. Three biological replicates were performed for each treatment. Different letters on the columns indicate a significant difference at *p* < 0.05.

In this study, six DEGs considered to be closely related to regulation of the auxins were identified (Table S2). To independently confirm the expression levels obtained from the RNA-seq data, six auxin-related DEGs were chosen to perform qRT-PCR analysis. As shown in Figure 8, the expression patterns of *IAA1*, *IAA11*, *AUX*/*IAA29*, *GH3.1* (*CSS0046770*), *GH3.5*, and auxin transport carrier gene (*CSS0041860*) were highly consistent with the transcriptome data, which suggests that the RNA-seq data are credible.

## 3. Discussion

Exogenous plant growth regulators are typically applied to induce the growth and development of plant explants and root formation of cutting seedlings. In the present study, IBA and NAA (auxins) were used to induce AR formation in tea plant cutting calluses, and the transcriptome and qRT-PCR results revealed that many genes, especially the auxin-related genes, were significantly differentially expressed under auxin treatment conditions. Auxin has been well known to be the central regulator that controls adventitious rooting [21,22]. During the AR formation period, many signaling-related genes were the most important molecular bases for the initiation of AR in the plants [23]. Among them, the Aux/IAA genes responding to auxin induction were considered to be the vital regulatory genes in the auxin signaling pathway [24]. As Poutrain et al. reported, the expression of early auxin-related gene *CrIAA1* can be dramatically induced by auxin treatment via a feedback mechanism in Catharanthus roseus [25]. However, Gan et al. found that *CsIAA1*, *CsIAA3*, *CsIAA6*, *CsIAA8*, *CsIAA10*, *CsIAA11*, and other Aux/IAA genes were inhibited by IAA in cucumber [26]. Nam et al. found that the nucleo-cytoplasmic distribution of the negative regulators of auxin signaling *IAA12* and *IAA19* regulates lateral root development under different abiotic stress conditions, and CONSTITUTIVE EXPRESSOR OF PATHOGENESIS-RELATED GENES 5 (CPR5) could selectively mediate the cytoplasmic translocation of *IAA12*/*19* [27]. Under abiotic stress conditions, CPR5 transcripts were obviously down-regulated, which will lead to the accumulation of nucleus-localized *IAA12/19* in the root elongation zone and, thus, inhibit lateral root development. In the present study, we found that *IAA1*, *IAA11*, *AUX*/*IAA4*, and *AUX*/*IAA29* in tea plant cutting calluses were up-regulated by IBA and NAA treatments. Among them, three genes were induced by exogenous auxin at all four treatment time points except for *IAA11*, indicating that these four Aux/IAA genes may be the core auxin-related genes that are involved in the auxin signaling pathway and promote AR formation.

Many researchers have also reported that TF genes play critical roles in AR formation. In Arabidopsis, a member of the AP2/ERF family gene, *RAV1*, participated in a branch pathway downstream to ARR1 that controls the expression of CRF1 to promote cytokinin action during primary root development [28]. A zinc finger protein encoding gene LATERAL ROOT PRIMORDIA 1 (*LRP1*) was highly expressed in the lateral and AR primordia of Arabidopsis [29]. A MYB TF gene, *MYB77*, played a role in auxin signal transduction through the modulation of known auxin-inducible genes; the mutation of *MYB77-1* inhibited the expression of all auxin-inducible genes [30]. *A* WRKY gene, *WRKY42*, was required for the activation of ROOT HAIR DEFECTIVE 6 (RHD6) to promote cold-induced ROOT HAIR (RH) expansion [31]. A NAC family gene, *NAC1*, was induced by wounding and functioned in promoting root rip emergence [32]. In the present study, we found 18 *zinc finger protein*, 8 *MYB*, 6 *WRKY*, and 9 *NAC* TF genes were up-regulated by exogenous auxin treatment, suggesting that these genes can be positively induced by exogenous auxin and are vital for AR formation and development. In addition, ARF transcription factors may play important roles in auxin-responsive molecular processes, which mediate auxin signaling at the transcriptional level by regulating the expression of auxin-responsive genes [33]. For example, *ARF6* and *ARF8* were reported to positively stimulate adventitious rooting by inducing the expression of *GH3*.3, *GH3.5*, and *GH3.6* genes [34,35]. Two ARF genes, *LkARF7* and *LkARF19*, from L. *kaempferi* overexpressed in poplars could promote adventitious root formation; *LkARF19’s* regulation of adventitious root formation mainly occurred by forming a heterodimer according to interactions with the DEAD-box ATP-dependent RNA helicase 53-like protein [36]. Chaudhary et al. found that many Aux/IAA and ARF components of T. *aestivum* specifically mediated axis formation, meristem commitment, and other re-differentiation processes [37]. In this study, we found *ARF5* and *ARF9* were up-regulated by exogenous IBA and NAA treatments; correspondingly, the expression levels of *GH3.1* and *GH3.5* were increased in WT12 vs. HT12, WT24 vs. HT24, and WT48 vs. HT48, suggesting that *ARF5* and *ARF9* may positively regulate the expression of *GH3.1* and *GH3.5* under IBA and NAA treatment conditions. In addition to mediating the expressions of *GH3* family genes, previous research also found that *ARF7* and *ARF19* could mediate adventitious root formation by positively activating the transcription of LBD genes [38]. During the AR development of Arabidopsis, the expressions of *LBD16* and *LBD18* were regulated by *ARF7* and *ARF19* [39,40]. In addition, *ARF7*, *ARF9*, and their downstream LBDs genes were obviously up-regulated by auxin treatment in blueberry, indicating that they may play important roles in the AR formation of blueberry [41]. In a study of *Camellia sinensis* cv. ‘ShuChaZao’, a total of 13 *CsLBD* genes were mainly expressed in roots, of which *CsLBD37_1/2/3*, *CsLBD38*, *CsLBD40*, and *CsLBD41_1* showed the highest expression levels in roots [42]. Here, we found *CsLBD39_2*, *CsLBD40*, and *CsLBD41_1* were up-regulated, which is consistent with the result of the previous study. In summary, the present results suggest that exogenous auxin treatment may induce the expressions of *ARF5* and *ARF9*; furthermore, *ARF5* and *ARF9* will promote the transcription of downstream target genes, such as LBD (*CsLBD39_2*, *CsLBD40*, and *CsLBD41_1*) and *GH3* (*GH3.1* and *GH3.5*), to establish adventitious root blasts with nuclear migration and control free IAA levels, respectively. Finally, they will further promote AR primordia generation and regulate AR formation and development. Additionally, SUPERROOT2 (SUR2)-encoded cytochrome P450, *CYP83B1*, blocks the synthesis of indole glucosinolates by oxidizing indole-3-acetaldoxine (IAOx), thereby elevating endogenous IAA levels and promoting AR formation [43]. Moreover, Xiao et al. found that calmodulin also participates in AR formation by modulating IAA content in poplars; overexpression of *PdeCML23-1* could enhance the accumulation of the active form of phytohormone IAA in transgenic lines [44]. In this study, we found fourteen CYP450 and five CML genes were up-regulated by exogenous auxin treatment, suggesting that the high expression of these genes may function to promote the accumulation of IAA. Thereafter, the high content of endogenous IAA may promote the expression of ARFs following the activation of downstream target genes, such as LBD and *GH3*, to generate AR primordium.

In many plant species, it has been reported that many GRAS family members, such as SCR, SCL, and SHR proteins, are responsible for the establishment of an embryonic root meristem [45,46,47,48]. Among them, SCR was reported to participate in cell division, differentiation, and cell homeostasis during the root meristem establishment of *Arabidopsis thaliana* [49]. After IBA treatment, *CsSCL1* changed the genetic expression patterns of cambial cells and derivative cells, which were then able to establish a new program and formed AR [50]. In juvenile rooting-competent cells of black walnut cuttings, the expression of *SCL* displayed a 23- to 24-fold increase and the expression of *SHR* displayed a 2- to 4-fold increase, indicating that *SCL* and *SHR* are important for AR primordium generation [51]. In this study, the expressions of *SCR* and *SHR* genes were not significantly changed, but *SCL* genes were up-regulated by exogenous auxin treatment, which were differentially expressed after 6 h of auxin treatment; only *SCL21* was up-regulated in all pairwise comparisons, implying that exogenous application of auxin induced up-regulated expression of *SCL* genes to promote division and differentiation of cambium cells and derived cells and AR formation.

## 4. Materials and Methods

### 4.1. Plant Material and Sample

The softwood cuttings of *Camellia sinensis* cv. ‘ShuChaZao’ were inserted into the seedling pool on 5 November 2020. Management of cutting seedlings was performed using the technical regulations of tea plant softwood cutting management (SDNYGC-1-4071-2018). The experiments were conducted at the North Tea Research Institute of Wulian County, Rizhao City, Shandong Province, China. On 26 April 2021, the cuttings with only calluses and no adventitious roots were selected and divided into two groups (80 cuttings per group). For the hormone treatment group (HT), one group of cutting seedlings above 10 cm was infiltrated in 50 mg/L IBA + 100 mg/L NAA of 5 L solution for 6 h; at the basic rooting agent ratio, IBA:NAA = 1:2 had the highest rooting rate after 6 h [52]. The other group of cutting seedlings was simultaneously infiltrated into distilled water for 6 h and served as the distilled water treatment group (WT). After 6 h of infiltration, all cutting seedlings were removed from the solution and placed into the seedling pool. In order to clarify the dynamic response of callus tissue to exogenous auxin treatment, HT and WT were sampled at 6 h, 12 h, 24 h, and 48 h after the above two treatments. The HT samples were named as HT6, HT12, HT24, and HT48, and the WT samples were named as WT6, WT12, WT24, and WT48. Three biological replicates were performed for each treatment. The obtained samples were immediately frozen in liquid nitrogen and stored at −80 °C for transcriptome analysis.

### 4.2. cDNA Library Construction, ONT RNA-Seq, Quality Control, and Function Annotation

Total RNA was isolated from the three samples using the RNA prep Pure Plant Kit (Tiangen, Beijing, China). Eight cDNA libraries (each sample with concentration > 20 ng/μL) were built using the PCR-cDNA Sequencing Kit (SQK-PCS109) and PCR Barcoding Kit (SQK-PBK004) and sequenced on the Nanopore PromethION platform. After base calling through Guppy software in the MinKNOW2.2 software package, the ONT RNA-Seq raw reads in fast5 format were converted to fastq format, filtering the low-quality (length less than 500 bp, Qscore less than 6) sequences. The cleaned sample data were aligned to the ribosomal RNA sequences in the database using the alignment tool BLAST. Ribosomal RNA was discarded after mapping to the rRNA database. Low-quality reads, short reads, and reads with adaptors were discarded to obtain clean data. Clusters of transcripts were obtained after mapping to a reference genome of R. *Camellia sinensis* (CSS_ChrLev_20200506.gff3.gz) using Mimimap2 [53], and consensus isoforms were obtained after polishing within each cluster using the Pinfish package. All unigenes were annotated using BLASTx search against the NCBI, Swiss-Port, KEGG, and KOG/COG databases [54,55].

### 4.3. GO and KEGG Pathway Enrichment Analyses

Differential expression analysis of the two groups was performed using the DESeq2 R package (1.6.3). DESeq2 provide statistical routines for determining differential expression in digital gene expression data using a model based on the negative binomial distribution. The resulting *p* values were adjusted using the Benjamini and Hochberg’s approach for controlling the false discovery rate. Genes with a FDR < 0.01 and foldchange ≥ 2 found by DESeq2 were assigned as differentially expressed. The methods used for GO and KEGG pathway enrichment analysis of DEGs were performed based on the hypergeometric test and referenced by Manzoor et al. and Manzoor et al. [56,57]. For GO analysis, the DEGS were grouped into three parts—cellular component (CC), molecular function (MF), and biological process (BP)—according to the GO database. For KEGG analysis, the DEGs were clustered into different pathways based on the specific function of each gene. Then, analyses were performed.

### 4.4. Reverse Transcription of cDNA and Gene Expression Analysis

The total RNA of each sample was extracted by using a Biospin Plant Total RNA Extraction Kit (BSC65S1, BioFlux, Beijing, China) with the provided instructions. cDNA was synthesized by using the PrimeScriptTM RT Master Mix (Perfect Real Time) (RR036A, TAKRA, Beijing, China) kit, according to manufacturer’s instructions. *GAPDH* was used as the internal reference gene of the tea plants, and primers used for gene expression were designed with Primer premier 5.0. Six DEGs were selected and examined using qRT-PCR to validate the reliability of the RNA-Seq results. The primes are listed in Table S3.

### 4.5. Statistical Analysis

Cluster Profiler was used as a hypergeometric test method to perform enrichment analysis of the KEGG annotation results of genes. Curve tables were designed using Excel 2019. Prism 6 and Photoshop 2020 were used to process and integrate data tables and images.

## 5. Conclusions

**Figure 9:**
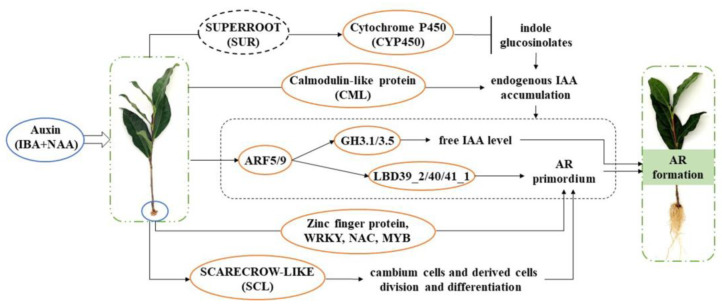
The gene modulation during AR formation of tea plant cuttings after exogenous auxin induction. The yellow box represents the up-regulated DEGs and the black dotted box represents unchanged genes. The arrows indicate the transition process from induction to formation of adventitious roots.

The root formation of tea plant cutting seedlings is a complex physiological process. Under exogenous auxin treatment conditions, we speculate that the increased expression of *CYP450* and *CML* genes may mediate the accumulation of IAA content in tea plant cutting seedlings (Figure 9). Moreover, both exogenous and endogenous auxin induced the expressions of ARF genes. Then, ARFs positively activated the transcription of LBD to promote the formation of adventitious root primordia and also positively regulated *GH3* genes to promote adventitious rooting by controlling free IAA levels. Furthermore, exogenous auxin also induced the expression of *SCL* genes, which controlled the division and differentiation of cambial cells and derivative cells to generate AR primordium. Meanwhile, some DEGs of TFs, such as zinc finger protein, *WRKY*, *NAC*, and *MYB*, participated in AR primordium generation. Finally, all of the above DEGs worked together to promote the formation of adventitious roots.

## Figures and Tables

**Table 1:** Function classification and expression patterns of auxin-related DEGs at different times.

#ID	log_2_FC				NR Annotation
	WT6 vs. HT6	WT12 vs. HT12	WT24 vs. HT24	WT48 vs. HT48	
CSS0016015	2.10	2.76	3.37	2.84	auxin-induced protein 22D
CSS0008143	2.46	3.95	4.11	2.58	auxin-responsive protein IAA1
ONT.21529	0.87	2.71	2.62	1.56	auxin-responsive protein IAA11
CSS0017030	2.11	3.33	3.29	2.65	auxin early response protein AUX/IAA4
CSS0019988	2.11	3.30	3.78	4.00	auxin early response protein AUX/IAA29
CSS0014406	4.08	5.73	5.67	6.80	GH3 auxin-responsive promoter
CSS0050062	4.17	5.34	5.61	6.66	GH3 auxin-responsive promoter
CSS0046770	4.74	7.50	6.57	6.24	auxin early response protein GH3.1
CSS0025132	5.15	7.90	6.68	6.34	auxin early response protein GH3.1
CSS0028487	0.76	2.34	1.66	2.27	auxin early response protein GH3.5
ONT.9850	7.48	6.66	4.69	1.44	auxin response factor 5
CSS0019949	1.17	1.53	1.27	−0.27	auxin response factor 9
CSS0041860	2.46	3.27	2.16	0.27	auxin transport carrier
CSS0050423	1.13	1.66	1.46	0.95	auxin transport carrier

## Data Availability

The transcriptomic data were deposited in the SRA database with accession number PRJNA896245.

## Supplementary Materials

The following supporting information can be downloaded at: https://www.mdpi.com/article/10.3390/ijms25158080/s1.
